# Multiplex CRISPR-Cas Genome Editing: Next-Generation Microbial Strain Engineering

**DOI:** 10.1021/acs.jafc.4c01650

**Published:** 2024-05-14

**Authors:** Se Ra Lim, Sang Jun Lee

**Affiliations:** Department of Systems Biotechnology and Institute of Microbiomics, Chung-Ang University, Anseong 17546, Republic of Korea

**Keywords:** multiplex genome editing, Cas nuclease, base editor, microbial production, guide RNA

## Abstract

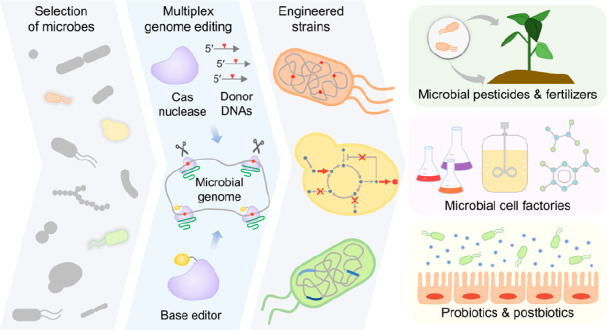

Genome editing is a crucial technology for obtaining desired phenotypes in a variety of species, ranging from microbes to plants, animals, and humans. With the advent of CRISPR-Cas technology, it has become possible to edit the intended sequence by modifying the target recognition sequence in guide RNA (gRNA). By expressing multiple gRNAs simultaneously, it is possible to edit multiple targets at the same time, allowing for the simultaneous introduction of various functions into the cell. This can significantly reduce the time and cost of obtaining engineered microbial strains for specific traits. In this review, we investigate the resolution of multiplex genome editing and its application in engineering microorganisms, including bacteria and yeast. Furthermore, we examine how recent advancements in artificial intelligence technology could assist in microbial genome editing and engineering. Based on these insights, we present our perspectives on the future evolution and potential impact of multiplex genome editing technologies in the agriculture and food industry.

## 1 Introduction

Living organisms develop and change according to the organism-specific genetic information programmed in the genome. Genome editing is an essential technology for altering genetic information in order to acquire desired phenotypes. By observing the genotype–phenotype changes, it is possible to study the principles of life phenomena, such as understanding the function of specific genes and verifying the interactions between genes. In addition to basic research, genome editing technology enables the development of environmental stress-resistant seeds,^1^ bioproduction of food additives and cosmetic ingredients,^2^ creation of animal models,^3^ and gene therapy.^4^

**Figure 1:**
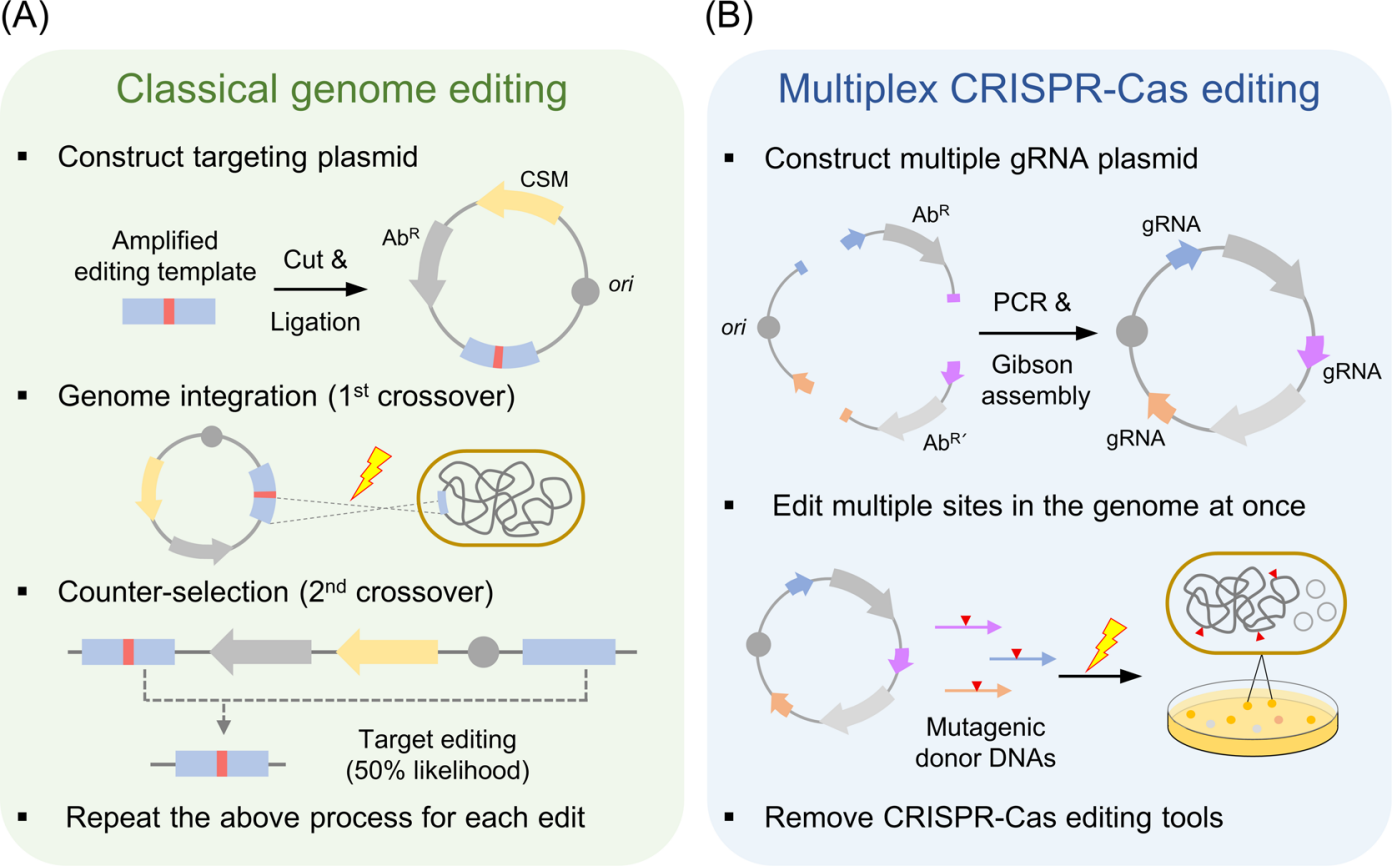
Procedures of classical genome editing and multiplex CRISPR-Cas genome editing methods. (A) The classical genome editing approach requires multiple suicide vectors, with two crossover events necessary to achieve a single target edit. (B) The multiplex CRISPR-Cas genome editing method uses a singular plasmid encoding multiple guide RNAs, enabling the attainment of desired multiple edits in a single mutagenesis round. Ab^R^, antibiotic resistance marker; CSM, counter-selectable marker; *ori*, the origin of replication.

Since the development of the clustered regularly interspaced short palindromic repeats (CRISPR)-CRISPR-associated proteins (Cas) technology, the field of genome editing has been advancing rapidly. The CRISPR-Cas system functions like a pair of genetic scissors, capable of cutting the target nucleic acid sequence by altering the target recognition sequence (TRS) of CRISPR RNA (crRNA).^5^ Unlike zinc-finger nucleases (ZFNs) and transcription activator-like effector nucleases (TALENs) that require protein engineering for recognition of wanted target DNA sequences, crRNA can be easily designed and modified.^6^ This flexibility has enabled the wide use of CRISPR-Cas technology in genome editing of almost all organisms, from bacteria to yeast, fungi, plants, insects, animals, and humans.^7−13^ To expedite genome editing, the multiplex editing method is being employed, enabling simultaneous editing at two or more loci in the genome (Figure 1). This approach reduces repeated plasmid integration, recombination processes, cost, and labor.

**Figure 2:**
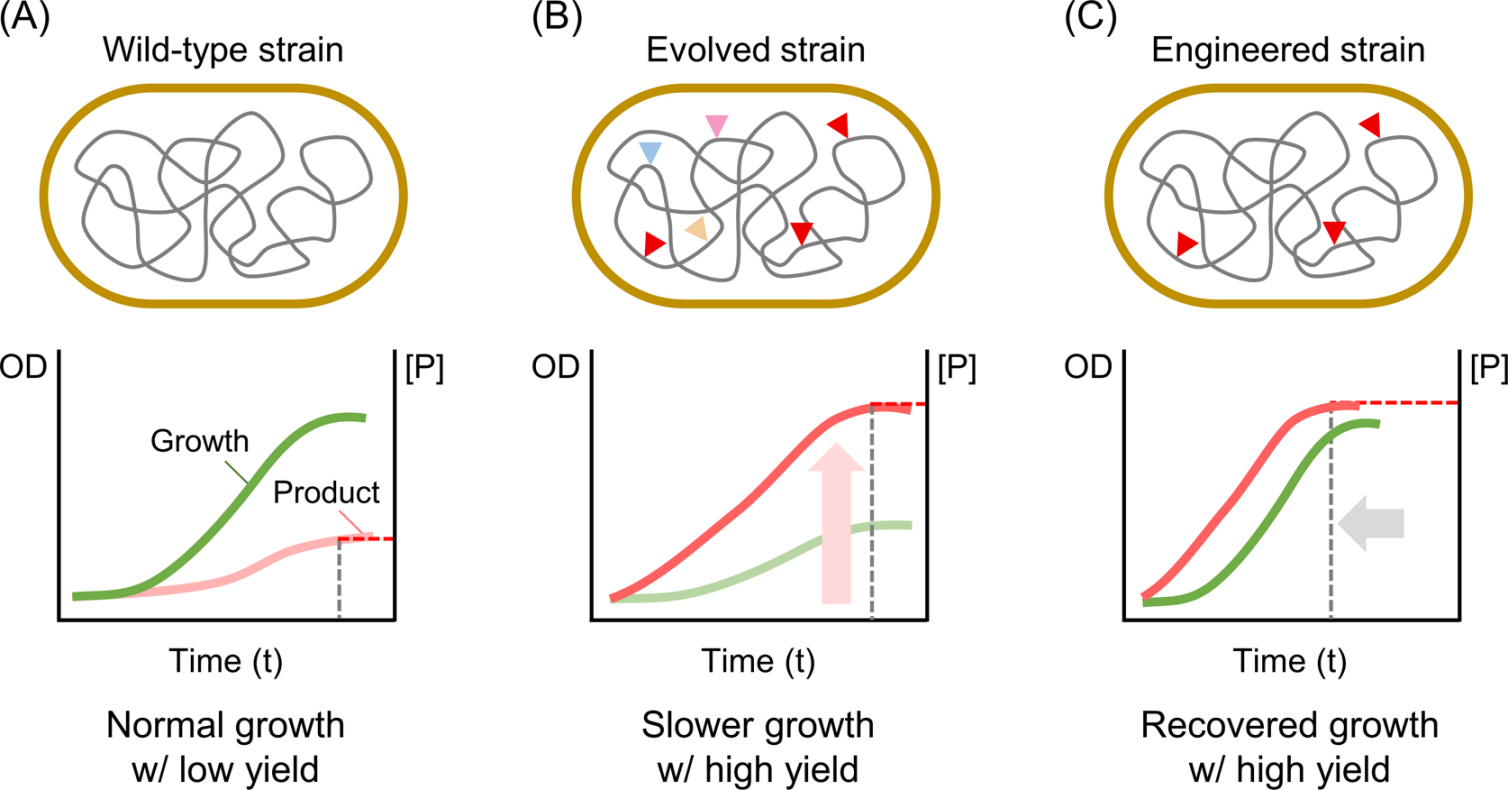
Microbial strain engineering for higher productivity through multiplex genome editing. (A) The wild-type strain showed a low yield of product ([P]/OD). (B) Evolved strain showed higher yield but slower growth, probably due to many nonspecific random mutations in the genome. (C) Transfer of beneficial mutations to new background or removal of unwanted mutations in the evolved cells, resulting in higher productivity ([P]/OD*t) in engineered cells. OD, optical density; [P], yield of product.

Multiplex genome editing is highly efficient in microbial strain engineering. For instance, microbial strains with increased production of target products are obtained through random mutagenesis or directed evolution (Figure 2A,B). Random mutagenesis often leads to the accumulation of unwanted mutations unrelated to the productivity, thereby decreasing the performance of the evolved strain.^14^ Unnecessary mutations can be restored to the wild-type, or necessary mutations can be transferred to another background to obtain a new strain with improved productivity like inverse metabolic engineering (Figure 2C).^15^ By using multiplex genome editing in this process, the new strain can be produced in a time- and cost-efficient manner. Most of the mutations that occur through chemical mutagenesis and adaptive evolution are point mutations;^16,17^ therefore, precise modification from mutations to the desired sequences requires accurate multiplex genome editing technology.

In this review, we summarize the current status of multiplex genome editing using CRISPR-Cas technology in bacteria and yeast. Our main emphasis lies in discussing editing resolution and the selection of suitable tools for multiplex editing. Additionally, we discuss the biochemical production in strains engineered through multiplex CRISPR-Cas genome editing. Lastly, we explore the integration of artificial intelligence (AI) with CRISPR-Cas genome editing tools and anticipate the future development of multiplex genome editing for practical applications, including agriculture and food production.

## 2 CRISPR-Cas-Mediated Genome Editing Methods

**Figure 3:**
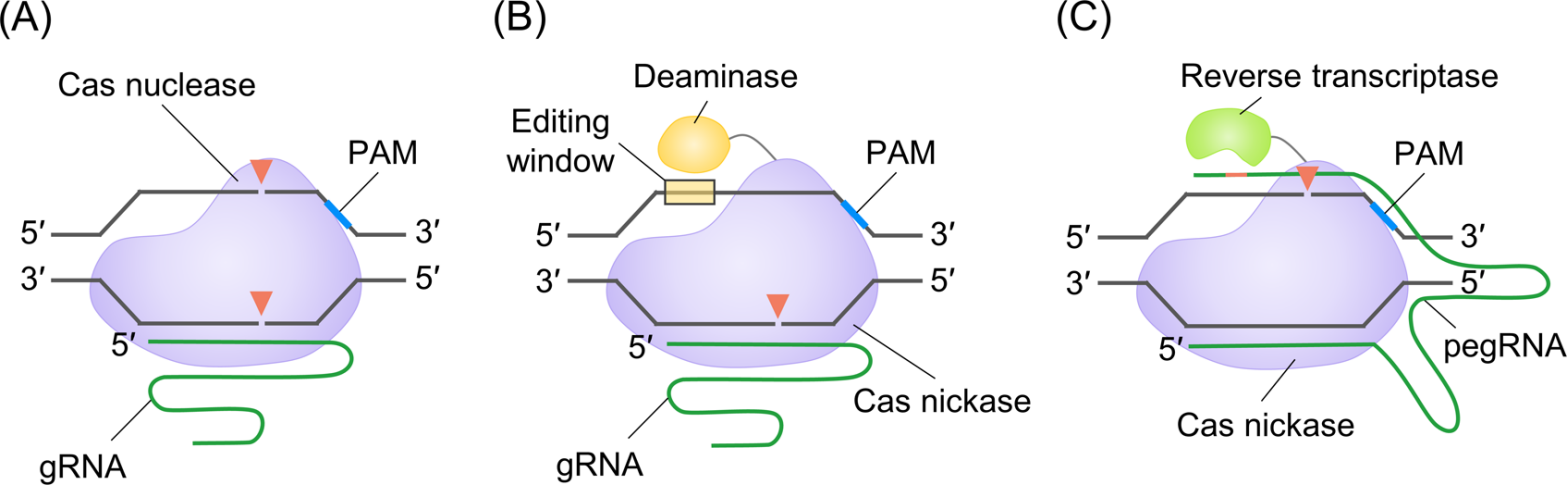
Genome editing tools derived from the CRISPR-Cas system. (A) The Cas nuclease introduces double-stranded breaks at specific DNA target sites, which are subsequently repaired via non-homologous end joining (NHEJ) or homology-directed repair (HDR), facilitating targeted genomic alterations. The guide RNA (gRNA) directs the Cas nuclease to the target site. (B) The base editor consists of a Cas nickase that induces single-stranded DNA breaks, coupled with a deaminase enzyme. This enzyme catalyzes the deamination of target nucleotides within a defined editing window, leading to precise base pair substitutions. (C) The prime editor comprises a Cas nickase, a reverse transcriptase, and a prime editing guide RNA (pegRNA). This system uses the pegRNA as a template to introduce desired mutations directly into the target DNA sequence.

In the CRISPR-Cas system, the guide RNA (gRNA) recognizes the target sequence, while the protospacer-adjacent motif (PAM) sequence is recognized by Cas nuclease. Subsequently, the gRNA-Cas nuclease complex cleaves the phosphodiester bonds of the DNA target, resulting in a double-strand break (DSB) (Figure 3A).^5^ When DSB is repaired by non-homologous end joining (NHEJ), then all cells are not repaired identically. Random insertions and deletions (indels) can occur during NHEJ.^18^ To introduce specific genetic modifications such as point mutations, small modifications, and large indels, a donor template DNA should be provided so that the DSB is repaired through homology-directed repair (HDR).^19^

Base editors were developed to edit targets without causing DSB.^20,21^ A base modifying enzyme such as deaminase is linked to a Cas9 protein moiety, either catalytically deactivated Cas9 nuclease or Cas9 nickase (Cas9n) (Figure 3B). In addition to Cas9, the Cas12 family has been employed in the development of base editors.^22^ Base editors facilitate nucleotide substitutions within the editing window through the action of adenine or cytosine deaminases. Efforts to increase editing efficiency have continued through the utilization of deaminase variants and Cas9 variants obtained via laboratory evolution.^23−25^ Recently, dual base editors have been used to perform both adenine and cytosine conversions.^26−28^ Due to their lack of causing DSBs, base editors exhibit low cytotoxicity and offer the advantage of not requiring donor template DNA, thus making them widely used in various microbes.^29^

A prime editor, which utilizes RNA as a template, has been developed, enabling precise editing to introduce all types of point mutations (Figure 3C).^30^ The reverse transcriptase linked to Cas nickase forms a complex with the prime editing guide RNA (pegRNA) and facilitates editing on the nontarget strand. Although prime editors have the advantage of being capable of installing any base-to-base changes, they are not widely used in multiplex genome editing. The reasons seem to be that prime editor requires a relatively complex pegRNA design and has a lower editing efficiency.^31^ Therefore, we have summarized the multiplex editing of microbial genomes achievable with Cas nuclease and base editors.

## 3 Multiplex Microbial Genome Editing by Cas Nuclease

By introducing multiple gRNAs and Cas nuclease into a cell, multiple targets in the genome can be edited simultaneously. The first attempt at multiplex genome editing in microbes using CRISPR-Cas technology was made in *Streptococcus pneumoniae*, simultaneously knocking out two genes with 75% efficiency.^32^ Subsequently, it was reported that two or three genes could be simultaneously edited, with efficiencies of 43% and 19%, respectively, in the eukaryotic microorganism *Saccharomyces cerevisiae*.^33^ There were many cases of knocking out genes by deletion in bacteria^34^ and integrating several kilobase-heterologous genes in yeast.^35^

In the plant pathogenic bacterium *Xanthomonas oryzae*, a crRNA array method has been proposed to simultaneously and efficiently knock out two virulence genes by overexpressing the proteins involved in NHEJ such as Ku and LigD.^36^ In yeast, there have been reports that multiple sites can be edited simultaneously by introducing identical synthetic gRNA binding sites into the genome^37−39^ or by cutting with gRNA recognizing common sequences among different genes.^40^ In *Ogataea thermomethanolica*, *Ogataea parapolymorpha*, *Komagataella phaffii*, *Scheffersomyces stipitis*, and *Yarrowia lipolytica*, which have a preference for NHEJ, two to four targets can be knocked out without donor template DNAs.^41−46^

While editing the genome at a resolution of 2–4 nt in bacteria was often reported (Table 1), cases of single-nucleotide-level multiplex genome editing were hard to find.^47,48^ In yeast, a few cases of multiplex editing at a single-nucleotide resolution are known.^49,50^ Accuracy of editing is important for correcting frameshift mutations, introducing point mutations, changing amino acid residues in polypeptide chains, and optimizing ribosome binding sites (RBSs). However, the CRISPR-Cas system, with its mismatch tolerance, can cleave both edited and unedited targets, leading to the death of edited cells and hindering the negative selection of single-nucleotide-edited cells.^51^ One approach to overcoming this inherent characteristic of the CRISPR-Cas system is by using maximally truncated single-guide RNAs (sgRNAs) to edit multiple targets at the single-nucleotide level simultaneously.^48^

**Table 1:** Resolution of Multiplex Genome Editing in Microorganisms Using Cas Nuclease or Nickase^a^

resolution (nt)	no. of targets	efficiency (%)	species	type of Cas nuclease	how to express gRNAs in plasmid	donor template DNA	description	ref
1	3	13.3	*Saccharomyces cerevisiae*	Cas9-NG	polycistronic sgRNA array	plasmid	single-nucleotide editing using gRNA-tRNA array and Cas9-NG	(49)
1	3	9	*Escherichia coli*	Cas9	separate sgRNA cassettes	ssOligo	single-nucleotide editing using 5′-end-truncated sgRNAs	(48)
1	3	3.7	*Corynebacterium glutamicum*	Cas12a	polycistronic crRNA array	ssOligo	CRISPR-Cas12a-RecT system	(47)
1	2	25	*S. cerevisiae*	Cas9	Separate sgRNA cassettes	dsOligo	simultaneous gene deletion, integration, and point mutation	(50)
2	2	88	*E. coli*	Cas9	separate sgRNA cassettes	ssOligo	overexpression of RecX to inhibit RecA	(88)
2	2	70	*E. coli*	Cas9	tandem monocistronic sgRNA array	ssOligo	overexpression of RecX to inhibit RecA	(87)
2	2	60	*E. coli*	Cas12a	polycistronic crRNA array	ssOligo	CRISPR-Cas12a-based double-plasmid system	(91)
3	6	n/d	*Bacillus subtilis*	Cas12a	polycistronic crRNA array	PCR product	overexpression of protein that promotes HDR and post-transformation incubation	(52)
3	5	100	*S. cerevisiae*	Cas9	tandem monocistronic sgRNA array	dsOligo	efficient construction of multiple gRNA vectors by the USER-cloning method	(151)
3	3	23	*E. coli*	Cas9	polycistronic sgRNA array	ssOligo	λ-red and ssOligo-mediated metabolic engineering	(152)
3	2	40	*C. glutamicum*	Cas9	tandem monocistronic sgRNA array	ssOligo	two-plasmid-based CRISPR-Cas9 system and a simplified cotransformation method	(153)
4	3	65	*B. subtilis*	Cas9n	tandem monocistronic sgRNA array	plasmid	enhancing HDR efficiency by *ligD* knockout	(58)
4	2	4	*Komagataella phaffii*	Cas9	polycistronic sgRNA array	PCR product	screening for gene knockouts by gel electrophoresis	(154)

a Note: n/d, not determined; ssOligo, single-stranded oligonucleotide; dsOligo, double-stranded oligonucleotide; HDR, homology-directed repair; Cas9n, Cas9 nickase.

During multiplex editing using Cas nucleases, the cell cannot survive due to DSB if one target in the genome remains unedited. Therefore, as the number of targets increases, the survival rate and editing efficiency of the cells inevitably decrease. To the best of our knowledge, the maximum number of targets that have been simultaneously edited using Cas nuclease was reported in *Bacillus subtilis*, where stop codons were introduced into six genes.^52^ In *E. coli*, simultaneous deletions of 500 bp at four distinct genetic loci have been achieved with an efficiency of 40%.^53^ In yeast, the highest number of targets simultaneously edited stands at eight, accomplished through transfer RNA (tRNA)-mediated gRNA processing.^54^ Due to high homologous recombination efficiency in yeast, it is common to use a method that separately introduces a plasmid backbone and multiple gRNA fragments into the cell, enabling the *in vivo* assembly of a gRNA plasmid for multiplex editing.^50,55,56^

Although not commonly used, the application of Cas nickase, which induces only single-strand breaks (SSBs), can enhance the cellular repair process, thereby improving the likelihood of obtaining successfully edited cells.^57−59^ In *B. subtilis*, the efficiency of simultaneous editing at three targets was 19.5% with Cas9 and 49.0% with Cas9 nickase, with the latter also resulting in a higher cell survival rate.^58^

## 4 Multiplex Microbial Genome Editing by Base Editor

Base editors, unlike Cas nucleases, do not require donor template DNA and can recognize multiple genomic targets to simultaneously create mutations within the editing window. This technology is widely applicable to multiplex genome editing in various bacterial species (Table 2). To date, the highest number of target genes simultaneously edited was 17 in *Streptomyces coelicolor*, where a plasmid containing 28 sgRNAs was introduced into the cell through conjugation.^60^ The number of simultaneously edited targets varied among the edited colonies, with counts of 17, 10, and 9 targets. This variability was due to intermolecular recombination occurring between repeating sgRNA sequences, leading to a different number of sgRNAs in the plasmid within each colony. In *E. coli*, dCas9-cytosine deaminase (CDA) construct failed to obtain multiplex-edited cells.^61^ However, by attaching the *ugi* gene encoding uracil glycosylase inhibitor, the mutagenesis rate in the multiple sites was increased. Consequently, it was possible to simultaneously edit six genes with an efficiency of 87.5%. In *Bacillus subtilis*, when only weak promoter was employed for the expression of base editors, three to five target genes can be simultaneously edited.^62^

**Table 2:** Efficiency of Multiplex Genome Editing in Microorganisms Using Base Editor^a^

no. of targets	efficiency (%)	species	type of base editor	how to express gRNAs in plasmid	description	ref
17	n/d	*Streptomyces coelicolor*	CBE	polycistronic sgRNA array	Csy4-based sgRNA processing and stronger promoter of sgRNA array	(60)
6	87.5	*E. coli*	CBE	tandem monocistronic sgRNA array	use of a uracil DNA glycosylase inhibitor and a degradation tag	(61)
5	75	*B. subtilis*	CBE	polycistronic sgRNA array	decreasing the expression of the base editor	(62)
5	42	*Yarrowia lipolytica*	CBE	tandem monocistronic sgRNA array	increasing the expression of the base editor and gRNA copy number, and KU70 deletion	(63)
4	50	*Bacteroides thetaiotaomicron*	CBE	tandem monocistronic sgRNA array	single-plasmid-based multiplex genome editing	(155)
4	30	*Lactococcus lactis*	CBE	tandem monocistronic sgRNA array	enhancing editing efficiency by subculturing	(156)
4	n/d	*Xanthomonas oryzae*	CBE	polycistronic sgRNA array	CBE promoter optimization	(98)
3	100	*E. coli*	ABE, CBE	separate one sgRNA cassette and tandem two sgRNA cassettes	laboratory-evolved deaminase	(27)
3	45.6	*Shewanella oneidensis*	ABE, CBE	tandem monocistronic sgRNA array	post-transformation incubation	(28)
3	90	*Sinorhizobium meliloti*	ABE	tandem monocistronic sgRNA array	single-plasmid-based multiplex genome editing using the Golden Gate assembly method	(157)
3	87.5	*Mycobacterium smegmatis*	CBE	tandem monocistronic sgRNA array	StCas9 fusion protein with uracil DNA glycosylase inhibitor or uracil DNA glycosylase	(158)
3	35	*Pseudomonas putida*	CBE	tandem monocistronic sgRNA array	use of engineered deaminase	(95)
3	12.5	*C. glutamicum*	CBE	tandem monocistronic sgRNA array	use of engineered deaminase	(159)
3	6.7	*Rhodobacter sphaeroides*	CBE	tandem monocistronic sgRNA array	second base editor induction by streaking	(160)
2	100	*Staphylococcus aureus*	ABE	tandem monocistronic sgRNA array	use of engineered deaminase	(96)

a Note: n/d, not determined; CBE, cytosine base editor; ABE, adenine base editor; Csy4, a member of CRISPR-associated endonuclease; StCas9, Cas9 from *Streptococcus thermophilus*.

In yeast cells, multiplex genome editing is mainly performed by Cas nuclease, and there were not many cases with base editors (Table 2). It has been reported that up to five targets could be edited in a Ku70-deficient strain in *Y. lipolytica*.^63^ The efficiency of editing each gene individually exceeded 75%, but it significantly decreased to 16% when attempting to edit five genes simultaneously. By increasing the expression of gRNAs and base editors, the simultaneous editing efficiency was enhanced to 42%.

Although base editors were developed for precise nucleotide substitution, it has a problem with bystander effects.^64^ The bystander effect refers to cases where a base editor not only alters its specific single target nucleotide but can also unintentionally modify nearby nontarget nucleotides within the editing window. Therefore, it may not always be suitable for precise editing purposes. Base editors can efficiently generate mutant libraries for microbial strain engineering by inducing hypermutation in the target region.^65,66^

## 5 Strategies to Improve Multiplex Genome Editing Efficiency

Compared to single-target editing, several issues arise when trying to edit multiple targets simultaneously. In multiple gRNA arrays, the repetitive components frequently lead to problems with the *in vitro* array synthesis and *in vivo* stable expression.^67^ The level of Cas nuclease expression needs to be finely tuned due to the decreased cell survival ratio caused by DSBs occurring in multiple loci in the genome.^68^ The efficiency of donor template DNA introduction into cells should be increased when Cas nuclease is used. There have been attempts to address these issues to enhance the efficiency of multiplex genome editing. Successful strategies are examined in detail in the following sections.

### 5.1 Multiple gRNA Expression Methods

**Figure 4:**
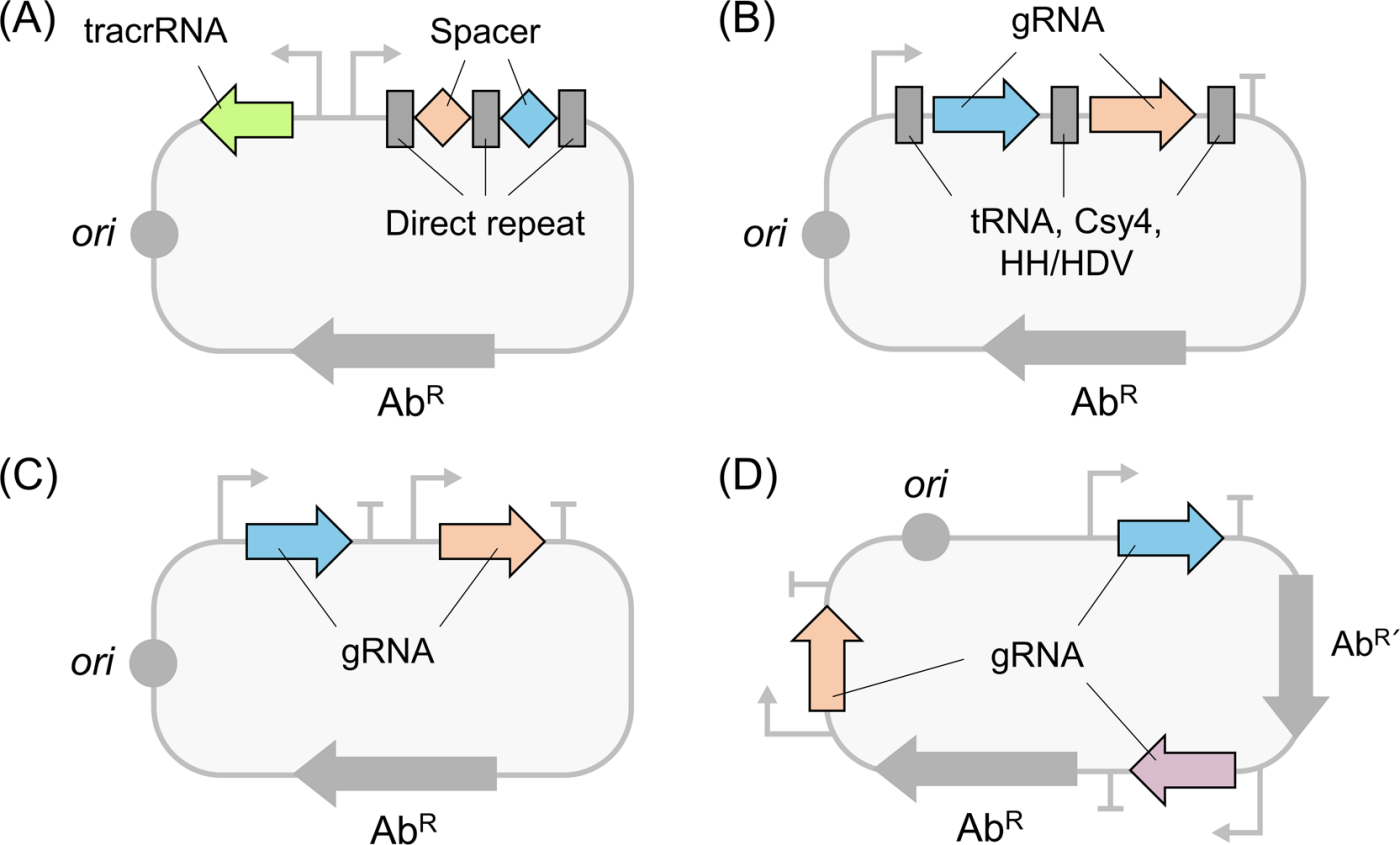
Various arrays of target-recognizing gRNAs in plasmid constructs. (A) tracrRNA and crRNA array, like the original CRISPR-Cas system.^32^ (B) Polycistronic gRNA array flanking tRNA,^54^ HH/HDV ribozymes,^82^ or Csy4 site^60^ for *in vivo* RNA transcript processing. (C) Tandem monocistronic gRNA array carrying each promoter and terminator.^72^ (D) Separated monocistronic gRNAs between *ori* and antibiotic resistance markers.^48^

The stable expression of multiple gRNAs is one of the most crucial elements for efficient multiplex genome editing. It is more efficient to express multiple gRNAs on a single plasmid than to transform multiple gRNA plasmids recognizing different targets.^46^ Various methods for producing multiple gRNA plasmids are introduced as follows. First, a method of separately expressing trans-activating CRISPR RNA (tracrRNA) and a crRNA array was employed similar to the natural CRISPR-Cas system, to prevent the loss of gRNA caused by recombination events between identical sequences repeated within each gRNA scaffold (Figure 4A).^32^

Second, to facilitate efficient gRNA processing from a polycistronic transcript, elements such as Csy4 or tRNAs can be inserted between gRNAs. Csy4 recognizes and cleaves at the Csy4 site.^69^ RNases P and Z can target 5′-leader and 3′-trailer sequences of tRNAs, respectively.^70^ In addition, ribozyme moieties such as hammerhead (HH) or hepatitis delta virus (HDV) can be also inserted (Figure 4B).^33^ Self-cleaving HH or HDV ribozymes act on the intramolecular cleavage site.^71^ Third, a tandem monocistronic gRNA array in a multiple gRNA plasmid can be conveniently created through the Golden Gate assembly or Gibson assembly method for the desired transcript levels of gRNAs (Figure 4C).^72^ This method can address the issue of transcriptional polarity, where downstream gRNAs in a long transcript may be expressed at lower levels.^73^

Repeated promoter and terminator sequences may lead to the loss of gRNAs in the multiple gRNA plasmid due to homologous recombination.^74^ Furthermore, the loss of gRNA caused by homologous recombination between repeated sequences in the gRNA array resulted in colonies edited only on a few of the multiple targets, reducing multiplex editing efficiency.^60,75−77^ A method to increase the heterogeneity within a polycistronic gRNA array by modifying the gRNA scaffold itself and using different tRNAs for processing has been proposed, but there are still many repeated sequences between each gRNA.^78^ Recently, gRNA cassettes were placed in the plasmid between the origin of replication (*ori*) and antibiotic markers in a plasmid to prevent the loss of gRNAs (Figure 4D).^48^

Next, to increase the copy number of multiple gRNAs within a cell, strong promoters are sometimes used,^45,79^ and high-copy-number plasmids are also used.^80^ Because the use of strong promoters can impose a growth burden, the utilization of a weaker promoter may be necessary.^81^ Therefore, it seems crucial to optimize the appropriate amount of gRNAs based on the microbial species and the number of targets by experimenting with plasmids of varying copy numbers or gRNA promoters of different strengths.

### 5.2 Regulation of DNA Repair and Recombination

DSBs induced by Cas nucleases at target sites are typically repaired through the NHEJ mechanism. This process often results in random indel mutations that can lead to the inactivation of the target gene.^18^ For precise editing of the target sequence, introducing donor template DNA into the cell allows for detailed editing via homologous recombination at the DSB site.^19^

Although suppression of the NHEJ pathway can affect normal cell growth,^83^ genes involved in the NHEJ mechanism, such as *ligD*, *ku70*, and *ku80*, can be knocked out to enhance the efficiency of homologous recombination in multiplex genome editing.^58,84,85^ There was an instance where the *E. coli* RecA protein was overexpressed to boost homologous recombination efficiency during CRISPR-Cas9-mediated multiplex pathway engineering.^86^ Conversely, there were reports that overexpression of RecX, which inhibits RecA activity, can effectively improve the efficiency of CRISPR-Cas9-optimized multiplex genome editing.^87,88^

To increase homologous recombination of the donor template DNA, phage-derived recombinase can be overexpressed. In addition to the widely used λ-red protein^89^ or RecET derived from Rac prophage,^90^ efforts are underway to identify new recombinases that can further increase editing efficiency.^91,92^ Additionally, there have been reports that overexpression of proteins, such as mutated NgAgo (*Natronobacterium gregoryi* Argonaute protein without enzyme activity),^52^ AtpD (β-subunit of ATP synthase),^93^ and hBrex27 (exon 27 domain of human BRCA2)^94^ can increase the efficiency of homologous recombination, thereby improving the efficiency of multiplex genome editing.

### 5.3 Use of Engineered Editing Tools and Optimization of Recovery Conditions

A Cas9 nuclease variant obtained during its cloning process could be used to enhance the efficiency of multiplex editing.^80,82^ Using a deaminase unit (e.g., APOBEC1, CDA1, TadA) obtained through protein engineering, higher editing efficiency could be achieved in multiplex editing.^27,95,96^ The application of AI-based structure prediction and protein engineering technologies to obtain and screen Cas nuclease and deaminase variants appears to have a positive impact on the multiplex genome editing field.^97^

Regulating the expression of Cas nuclease, Cas nickase, or the base editor is crucial to minimize their impact on cellular function. The most common approach is to optimize promoter strength^62,98^ There is also a method for direct control of Cas9 activity by expressing anti-CRISPR proteins.^93^ AcrII4 (anti-CRISPR protein) could prevent leaky expression of Cas9 during delivery of the Cas9 editing tools into *S. coelicolor*, increasing the survival rate of *S. coelicolor* cells and facilitating efficient multiplex genome editing.

Recovery conditions, such as duration and subculture, also seem to affect editing efficiency. Extending the post-transformation incubation period can improve editing efficiency.^28,52,99^ Additionally, subculturing of editing candidates is a common and effective strategy.^59,100,101^ Particularly when using Cas nickase, partial editing can lead to a mixed-genotype where only a few of the multiple targets are edited even if any sgRNA is not lost. However, cells with completely edited multiple targets could be obtained through the subculture process.^59^ Nonetheless, during the subculture process, spontaneous mutations and adaptive mutations can occur,^102,103^ potentially compromising the accuracy of the editing.

### 5.4 Other Strategies

As the number of targets increases, multiple copies of gRNAs compete for binding with the limited quantity of Cas protein expressed within the cell.^67^ If the expression of the Cas protein, which has a high affinity for nucleic acids, is increased unnecessarily, it will inevitably burden the cell.^104^ Therefore, in multiplex genome editing, the endogenous CRISPR-Cas system could be used to alleviate the toxicity of heterologous Cas proteins.^101,105−108^ Other types of CRISPR-Cas systems, such as Cas12a (also known as Cpf1, 1200–1500 amino acids^109^) could be used in organisms like *Corynebacterium glutamicum* and cyanobacteria, where editing is challenging due to the toxicity of Cas9 (900–1700 aa^110^).^111^ Furthermore, the easily deliverable miniature Cas12f (400–700 aa^112^) and Cas12j (700–800 aa^113^) could also be used for multiplex genome editing.

For successful high-efficiency simultaneous editing of multiple targets, a deep molecular-level understanding of nucleic acid metabolism, such as DNA structure, repair, and recombination mechanisms, will be required. Among multiple targets, some may not be edited effectively, and three-dimensional (3D) genome topology data could also be helpful in selecting editing target loci and improving editing efficiency.

## 6 Application of Multiplex Microbial Genome Editing

When selecting microbial strains for multiplex genome editing, there are typically two approaches. The first approach involves choosing strains that have robust genome editing capabilities, making them suitable for integrating foreign genes.^114^ The second approach focuses on selecting strains that already exhibit the desired traits; these strains are then enhanced or upgraded using genome editing, but without introducing any foreign genes. In both scenarios, genome editing tools are essential for strain engineering. After the genome editing process, it is important to ensure that the editing tools are not left behind, as their presence could cause environmental or ecosystem issues.^115^

Techniques such as the use of counter-selectable markers and temperature-sensitive *ori* of plasmids allow for scarless editing that is indistinguishable from natural or chemical mutagenesis.^116,117^ Efficient use of genome editing tools in microorganisms and ensuring their removal post-editing will significantly advance the development of agriculture and the food industry. The following discussion will explore how CRISPR-Cas technology can be applied in the area of agricultural biologicals, strain engineering for microbial production, and improvement of probiotic strains, as well as the potential evolution of each field through multiplex genome editing technology.

### 6.1 Agricultural Microorganisms

Efforts are ongoing to reduce the use of synthetic pesticides and chemical fertilizers for eco-friendly agriculture and sustainable production, but for high productivity, it is not easy to prohibit the use of environmentally harmful chemicals. The performance of currently used biopesticides still needs to be improved. *Bacillus thuringiensis*, a representative biopesticide, plays dual roles in agriculture. Its spores contain an insecticidal crystal protein that eradicates pests feeding on crops, and the bacterium can enzymatically degrade the quorum sensing signals of plant pathogens, thereby protecting plants from bacterial infections.^118^ When *B. thuringiensis* is utilized on a large scale in agriculture, its crystal protein is vulnerable to degradation by ultraviolet rays. To mitigate this weakness, strains have been developed through genome editing using the CRISPR-Cas9 system that can produce melanin, enhancing their UV resistance.^119^

Biofertilizers are bacteria or fungi that can facilitate nitrogen fixation, plant growth hormone secretion, and promote plant nutrient absorption.^120^ If functional microbial strains that produce substances helpful for plant growth, such as auxin or naringenin, are created and used, from which a synergistic effect can be obtained.^121^ Genome editing tools have been developed in some rhizospheric bacteria, such as *Bacillus mycoides* and *B. subtilis*.^122^ Therefore, further research could be carried out to improve the performance of these strains. There have been few instances where multiplex genome editing has been applied to agricultural biologicals such as biopesticides or biofertilizers. However, it is anticipated that this technology will soon be widely utilized to develop strains with multiple functions and enhanced performance.

### 6.2 Cell Factories

The use of microorganisms as cell factories for the production of valuable substances will continue to increase.^123^ Considering the handling of toxic substances produced by chemical synthesis and the cost of refining byproducts, the production of food additives, nutrients, and antibiotics through microbial cell factories is much more eco-friendly. Typically, wild-type strains are not highly productive, but their productivity can be increased by expressing heterologous genes or modifying metabolic pathways. Currently, multiplex genome editing technology is primarily utilized to stably express heterologous genes by integrating them into the genome (Table 3). Antioxidants such as astaxanthin^124,125^ and β-carotene,^126,127^ which can be used as food additives, are being produced in *S. cerevisiae* through heterologous gene expression.

**Table 3:** Biochemical Production in Engineered Strains via Multiplex CRISPR-Cas Genome Editing^a^

target product	type of metabolic pathway modification	species (no. of multiplex-edited targets)	production level	ref
**Base Editor-Mediated Strain Engineering**				
glutamate	gene knockout	*C. glutamicum* (3)	4.3 g/L (3-fold increase)	(100)
naringenin	gene knockout	*Y. lipolytica* (4)	120 mg/L (2-fold increase)	(63)

protocatechuic acid	gene knockout	*P. putida* (9)	2.1 g/L	(161)
	gene knockout	*P. putida* (2)	264.9 mg/L (611.17% increase)	(95)

riboflavin	RBS optimization	*S. oneidensis* (3)	3-fold increase	(28)

**Cas Nuclease-Mediated Strain Engineering**				
*N*-acetylglucosamine	gene knockout	*B. subtilis* (6)	2.2 g/L (1.51-fold increase)	(52)

astaxanthin	integration of heterologous genes	*K. phaffii* (3)	n/d	(124)
	integration of heterologous genes	*S. cerevisiae* (3–5)	n/d	(125)

β-carotene	RBS optimization	*E. coli* (3)	212 mg/L (2.8-fold increase)	(152)
	integration of heterologous genes	*S. cerevisiae* (3)	n/d	(126)
	integration of heterologous genes	*S. cerevisiae* (3)	12.7 mg/L	(127)

*p*-coumaric acid	integration of feedback-resistant genes	*S. cerevisiae* (2)	4-fold increase	(162)
free fatty acids	gene knockout	*S. cerevisiae* (4)	559.52 mg/L (30-fold increase)	(54)
3-hydroxypropionic acid	integration of heterologous genes	*S. cerevisiae* (3)	195% increase	(163)
lactate	integration of heterologous genes	*S. cerevisiae* (4)	1.7 g/L	(79)
3-methylcatechol	integration of heterologous genes	*K. phaffii* (2–3)	n/d	(164)
6-methylsalicylic acid	integration of heterologous genes	*K. phaffii* (2–3)	n/d	(164)
mevalonate	gene knockout	*S. cerevisiae* (5)	1.5 mg/L (41.5-fold increase)	(151)
mogrol	integration of heterologous genes	*S. cerevisiae* (4)	5.9 mg/L	(94)
patchoulol	integration and optimization of heterologous genes	*S. cerevisiae* (3)	52 mg/L	(165)

protocatechuic acid	integration of heterologous genes	*S. cerevisiae* (3)	2.7 g/L	(84)
	integration of heterologous genes	*Kluyveromyces lactis* (3)	1.9 g/L	(84)

resveratrol	integration of heterologous genes	*Ogataea polymorpha* (3)	4.7 mg/L	(166)
riboflavin	RBS optimization	*B. subtilis* (3)	1.4 g/L (159% increase)	(58)

a Note: RBS, ribosome binding site; n/d, not determined.

If the pathway for the target metabolite is known, the yield can be increased by modifying the RBS or knocking out the genes involved in the formation of byproducts. In the case of RBS modification, changing even a single nucleotide could have a substantial impact on gene expression.^128^ An RBS library can be created using a base editor. For example, when targeting the RBS regions of genes involved in riboflavin biosynthesis, it was possible to obtain strains with improved riboflavin production among edited strains with various combinations of RBSs.^28^ Alternatively, the RBS sequences of lycopene biosynthesis genes could be randomly edited by a base editor.^129^ The advantage of this approach is that only two types of gRNAs are needed to target up to 10 different RBSs.

### 6.3 Probiotic Strains

In addition to the production of certain substances, bacterial strains could be developed to have specific features according to the purpose (e.g., a strain that expresses less extracellular protease,^130^ a strain in which five antibiotic biosynthetic gene clusters are knocked out^62^). For instance, it has recently been reported that two genes can be deleted simultaneously with over 90% efficiency in *Lacticaseibacillus paracasei*.^106^ It was possible to confirm the roles of two lactate dehydrogenase genes (*ldhD* and *ldhL*) in the stereospecific production of d- and l-forms of lactic acid in *L. paracasei*. This demonstrates the potential of multiplex genome editing tools to develop lactic acid bacterial strains at the laboratory level. When the editing of real-world probiotic targets becomes feasible, it may then become possible to engineer new strains capable of yielding more beneficial postbiotics.

## 7 Artificial Intelligence Technology on Genome Editing

AI technology is being utilized to more precisely and efficiently edit targets using CRISPR-Cas technology.^131^ It is widely used to predict the efficiency of gRNA (Table 4). CRISPR-Cas editing efficiency can be influenced by various factors, notably the genetic sequence and chromatin 3D structure.^132,133^ Particularly, efforts are also being made to predict genome editing efficiency by integrating 3D genomics research data.^134^ Prokaryotic chromatin structures undergo dynamic changes due to processes such as cell division and gene expression.^135^ gRNA design tools trained with eukaryotic organism data do not accurately predict efficiency when applied to bacteria.^136^ Therefore, tools have been developed for designing gRNAs for bacteria.^136−139^

**Table 4:** AI-Based gRNA Efficiency Prediction Tools for Microorganisms^a^

name of tool	species	type of Cas nuclease	description	ref
sgRNA-cleavage-activity-prediction	*E. coli*	SpCas9, eSpCas9	consideration of GG dimers and GC content	(136)
DeepSgRNABacteria	*E. coli*	SpCas9, eSpCas9	training on the differences between prokaryotes and eukaryotes	(137)
ssCRISPR	*E. coli*, *Pseudomonas* spp.	SpCas9, LbCas12a	design of strain-specific gRNA based on genome information	(138)
crisprHAL	*Citrobacter rodentium*, *E. coli*, *Salmonella enterica*	SpCas9, TevSpCas9	gRNA prediction by excluding gRNAs that hinder cell growth	(139)
DeepGuide	*Y. lipolytica*	SpCas9, LbCas12a	training of strain-specific *in vivo* cleavage profile and nucleosome occupancy data	(167)
crispRdesignR	*S. cerevisiae*	SpCas9	consideration of hairpin structure, GC content, and homopolymer (e.g., GGGG, TTTT)	(168)

a Note: SpCas9, Cas9 from *Streptococcus pyogenes*; LbCas12a, Cas12a from *Lachnospiraceae bacterium* ND2006; eSpCas9, An engineerd SpCas9 variant with high-fidelity (K848A/K1003A/R1060A);^169^ TevSpCas9, I-TevI nuclease domain–SpCas9 fusion protein;^170^ All source codes are available on https://github.com/.

When optimally designed gRNAs for Cas nuclease are used in base editor-mediated editing, the editing efficiency is often not high. Notably, specific bacterial design tools for base editors and prime editors have not yet been developed. A gRNA efficiency prediction tool suitable for base editors has been developed, allowing for the prediction of base-editing efficiency and outcome product frequencies in the human genome.^140^ For prime editors, a machine learning-based model has been developed to predict the editing efficiency of gRNA by considering factors such as the total length of the primer-binding site and editing template in pegRNA, as well as the GC ratio.^141^

AI-based protein structure engineering can also be used to enhance CRISPR editing tools to improve editing efficiency. Through the combination of a multidomain mutation library and the utilization of machine learning, the identified optimal Cas9 variant demonstrated a 7.5-fold increase in editing activity compared to the wild-type Cas9 nuclease.^142^ In another study, by calculating the expected editing efficiency from combining Cas9 variants with deaminase variants, it was possible to obtain an enhanced base editor with up to a 20-fold efficiency improvement.^143^

AI technology is being used in the field of metabolic pathway engineering to increase the production of valuable substances.^144^ Novel biosynthetic gene clusters in microbial genomes could be predicted,^145^ and the process of designing and evaluating synthetic promoters could be facilitated by AI-based methods.^146,147^ A deep learning-derived RBS–phenotype prediction model enabled the screening of only 3% of the RBS library, reducing experimental efforts while enhancing limonene productivity in *E. coli*.^148^ A machine learning model identified the optimal combination of promoters and terminators for each gene in the heterologous violacein biosynthesis pathway, resulting in a 2.4-fold increase in productivity in *S. cerevisiae*.^149^ In addition to fine-tuning the details of biosynthetic pathways to identify the optimal combination, the optimization of metabolic flux analysis is also becoming feasible with AI technologies.^150^ With the application of multiplex genome editing technologies, these approaches could quickly determine which target genes to select and how to edit their sequences for high yield and productivity in metabolite production.

## 8 Perspectives

Similar to employing robots and automated systems for the screening of microbial strains with desired characteristics, multiplex CRISPR-Cas genome editing technology offers significant labor and time savings compared to conventional genetic engineering techniques.

The integration of AI technology not only aids the CRISPR-Cas system in predicting optimal genetic targets for desired traits but also contributes to the enhancement of editing efficiency by designing the necessary gRNAs across various CRISPR-Cas editing tools. Furthermore, AI technology will also help overcome the limitations on the number of targets that can be edited at once and significantly reduce the experimental trials and errors when using multiplex genome editing techniques.

The CRISPR technology, with its modularity allowing for distinct recognition and cleavage, will continue to evolve in multiple directions beyond multiplex genome editing. If there are microbes with removable genetic tools and industrial potentials, they can soon be transformed into robust engineered strains using CRISPR-Cas integrative technologies. In the future, multiplex genome editing will become a pivotal technology for the sustainable advancement of the agricultural and food industries.

## Abbreviations

3D: three-dimensional
AI: artificial intelligence
Cas9: clustered regularly interspaced short palindromic repeats-associated protein 9
Cas9n: Cas9 nickase
CRISPR: clustered regularly interspaced short palindromic repeats
crRNA: CRISPR RNA
DSB: double-strand break
gRNA: guide RNA
HDR: homology-directed repair
NHEJ: non-homologous end joining
PAM: protospacer-adjacent motif
pegRNA: prime editing guide RNA
RBS: ribosome binding site
sgRNA: single-guide RNA
SSB: single-strand break
tracrRNA: trans-activating CRISPR RNA
tRNA: transfer RNA
TRS: target recognition sequence
